# Phosphate binder in dialysis: a cross-sectional study of patients' adherence and pill burden

**DOI:** 10.1590/2175-8239-JBN-2024-0075en

**Published:** 2025-01-20

**Authors:** Brunelle Bruna Scavello Coelho Ferezin, Luiza Karla Ramos Pereira de Araújo, Carolina Marquez Lima, Hugo Abensur, Benedito Jorge Pereira, Maria Aparecida Dalboni, Rosa Maria Affonso Moyses, Rosilene Motta Elias

**Affiliations:** 1 Universidade Nove de Julho, São Paulo, SP, Brazil.; 2 Universidade de São Paulo, Hospital das Clínicas, São Paulo, SP, Brazil.

**Keywords:** Phosphate Binder, P Binder, Chronic Kidney Disease, Dialysis, Hyperphosphatemia, Sevelamer, Calcium Carbonate

## Abstract

**Introduction:**

Phosphate (P) binders are among the most common medications prescribed to control P levels in patients with chronic kidney disease on dialysis. There is still a paucity of data on adherence to P binders with no comparison between dialysis modalities.

**Methods:**

We accessed factors associated with P binder adherence among patients on dialysis in an academic hospital. Adherence was calculated as the ratio between the number of pills taken per day as reported and the prescribed number of pills. Patients were considered non-adherent if adherence was at least 20% less or 30% more than prescribed.

**Results:**

Patients (N = 137) were young, mostly women, and on dialysis for a median time of 53 months. Sevelamer and calcium carbonate were prescribed as P binders to 70.8% and 10.2% of patients, respectively, with no difference across dialysis modalities (p = 0.839). P correlated with the number of pills prescribed (r = 0.368, p = 0.001) and the number of pills taken per day (r = 0.275, p = 0.001). Hyperphosphatemia was found in 52 patients (36.4%). Adherence to Ca carbonate and sevelamer was 100% and 68.4%, respectively. Non-adherent patients were women, younger, with higher serum albumin and urea, and lower serum calcium. Logistic regression showed that female sex (HR 3.30, 95% CI: 1.39–7.84, p = 0.007) and hemodialysis vs. peritoneal dialysis (HR 4.55, 95%, CI: 1.26–16.39, p = 0.021) remained independently associated with a non-adherence behavior.

**Conclusions:**

The current study suggests that strategies to increase adherence should be implemented. Whether phosphate binder adherence is associated with better outcomes deserves further investigation.

## Introduction

Patients with chronic kidney disease on maintenance dialysis (CKD5D) are at high risk of hyperphosphatemia, a complication that requires diet counselling, intensive dialysis, and phosphate (P) binders, which are among the most common medications prescribed to these patients. Indeed, treatment of hyperphosphatemia is burdensome, and half of the pills prescribed per day are usually P binders^1^. Clinical guidelines recommend lowering P toward normal ranges and restricting calcium-based P binders^2^. However, ongoing studies are challenging this classical concept^3^.

Medication adherence studies in the general population have shown that 50% of patients do not take their medications as prescribed^4,5,6^. According to the Dialysis Outcomes and Practice Patterns Study, only 45% of dialysis patients reported taking all their prescribed P binders during the previous month^7^. Haynes said that “increasing the effectiveness of adherence interventions may have a far greater impact on the health of the population than any improvement in a specific medical treatment”^4^. Since higher levels of serum P have been associated with increased cardiovascular risk and mortality^8^, adherence to the P binder is an important issue. This concept has been reinforced by the observation of an inverse relationship between P binder pill burden and serum P control^8,9,10^.

Although medication adherence in general is well documented in the literature, there is still a paucity of data on adherence to P binders. In addition, there is no comparison between dialysis modalities on this matter. Therefore, this study aimed to assess factors associated with P binder adherence among patients on assorted dialysis modalities (peritoneal dialysis, conventional hemodialysis, and short daily hemodialysis) in a single academic hospital in Brazil.

## Methods

This was a cross-sectional study that included adult patients (age ≥ 18 years) on maintenance dialysis at the Hospital das Clínicas HCFMUSP, Universidade de São Paulo, Brazil. Patients on either thrice-weekly hemodialysis (HD), short daily HD, or peritoneal dialysis (PD) were asked to participate. All patients on dialysis on the first day of July 2021 were invited to participate.

Clinical, laboratory, and demographic data were collected from charts and included age, gender, race, presence of diabetes, etiology of kidney disease, and dialysis modality. Biochemical variables of interest were serum albumin (RR 3.4–4.8 g/dL), total calcium (RR 8.4–10.2 mg/dL), alkaline phosphatase (reference range, RR 35–104 UI/L for women and 40–129 UI/L for men), phosphate (RR 2.7–4.5 mg/dL), parathyroid hormone (PTH) (immunoassay chemiluminescence, RR 15–65 pg/mL), and 25(OH)-vitamin D (chemoimmunoassay, RR 30–100 ng/dL). Hyperparathyroidism was defined as PTH > 300 pg/mL, hypovitaminosis D as levels < 30 ng/dL and hyperphosphatemia as levels > 5.5 mg/dL**.** Monthly routine laboratory results were evaluated. All analyses were performed by the same laboratory.

The Local Ethics Committee at the HCFMUSP has approved the study protocol (#45163715.4.0000.0068).

### Adherence

The self-reported adherence was determined by asking each patient the following question: “People miss taking their medication for several reasons. How many pills do you usually take per day?” We did not specify a time frame for response. The answers to these questions were used to calculate self-reported adherence using the equation: reported number of pills taken per day/prescribed number of pills as on label × 100. If a given patient indicated that he/she usually takes 4 tablets a day for a medication prescribed as 2 tablets a day, the self-reported adherence was calculated as 200%. Patients were considered non-adherent if self-reported adherence was at least 20% lower than prescribed or 30% higher than prescribed.

### Statistical Analysis

Continuous data are presented as mean ± standard deviation or median (25, 75 percentiles) as appropriate. Categorical variables are presented as frequency. Comparisons between adherent and non-adherent patients were performed using Student’s t-test or Mann-Whitney U tests for variables that were normally and not normally distributed, respectively. Logistic regression analysis was done to test independent factors associated with non-adherence. Independent variables, selected from the univariate analysis (p < 0.05), were introduced into the model one at a time and included age, calcium, dialysis modality (HD *vs.* PD), albumin, and urea. Since urea and albumin were collinear, they were not included in the same model. We considered p values < 0.05 significant. All analyses were performed using SPSS version 26 (SPSS Inc. Chicago, IL).

## Results

Adherence could be assessed in 137 of the 144 patients screened (95.1%). No patient refused to participate, 2 patients were transferred to another dialysis before the assessment and 5 patients were not included due to lack of understanding (2 mentally compromised and 3 who had their medication given by a third person who could not be reached during the study). As shown in Table 1, patients were relatively young, mostly Caucasian women, on dialysis for a median time of 53 months, with a low prevalence of diabetes. Most patients were on maintenance hemodialysis.

**Table 1:** Characteristics of patients

Variable	All N = 137	Non-adherent N = 33	Adherent N = 104	P
Female sex, n (%)	66 (48.2)	23 (69.7)	43 (41.3)	**0.005**
Diabetes, n (%)	22 (16.1)	17 (16.3)	5 (15.2)	0.871
Age, years	47 ± 17	42 ± 13	48 ± 17	**0.047**
Caucasian	100 (73.0)	76 (73.1)	24 (72.7)	0.979
Etiology of kidney disease, n (%)	20 (14.6)	5 (15.1)	15 (14.4)	0.899
Diabetes	17 (12.4)	3 (9.1)	14 (13.5)	
Nephrosclerosis	39 (28.5)	8 (24.2)	31 (29.8)	
Glomerulonephritis	16 (11.7)	5 (15.1)	11 (10.6)	
Interstitial disease	14 (10.2)	4 (12.1)	10 (9.6)	
SLE	32 (23.3)	9 (27.3)	23 (22.1)	
Other/unknown				
Dialysis vintage, months	50 (22, 92)	105 (71, 168)	50 (18, 84)	0.868
Dialysis modality, n (%)	89 (65.0)	27 (81.8)	62 (59.6)	**0.033**
Conventional 3 times/week	12 (8.7)	3 (9.1)	9 (8.7)	
Short daily	36 (26.3)	3 (9.1)	33 (31.7)*	
Peritoneal dialysis				
Serum albumin, mg/dL	4.0 ± 0.4	4.2 ± 0.3	4.0 ± 0.4	**0.048**
Total calcium, mg/dL	8.9 ± 0.8	8.7 ± 0.8	8.9 ± 0.8	**0.036**
Phosphate, mg/dL	5.3 ± 1.7	5.7 ± 1.9	5.2 ± 1.6	0.103
Hyperphosphatemia, n (%)	51 (37.2)	15 (45.5)	36 (34.6)	0.278
Alkaline phosphatase UI/L	104 (76, 154)	105 (71, 168)	104 (84, 139)	0.986
Parathyroid hormone, pg/mL	333 (193, 597)	333 (193, 585)	332 (165, 744)	0.740
Hyperparathyroidism, n (%)	76 (55.5)	17 (51.5)	59 (56.7)	0.558
25(OH)-vitamin D, ng/dL	33.6 ± 10.5	32.9 ± 10.7	33.8 ± 10.5	0.648
Hypovitaminosis, n (%)	48 (35.0)	12 (36.4)	36 (34.6)	0.849
Hemoglobin, g/dL	11.0 ± 1.7	10.9 ± 1.5	11.0 ± 1.7	0.698
Urea, mg/dL	43 (30, 95)	47 (31, 104)	38 (28, 50)	**0.046**
Creatinine, mg/dL	9.2 ± 3.0	10.0 ± 3.2	9.0 ± 2.9	0.060

Abbreviation – SLE: systemic lupus erythematosus. Notes – Data are expressed as mean ± SD or median (25, 75) unless otherwise specified. *Post-test p < 0.05.

**Figure 1:**
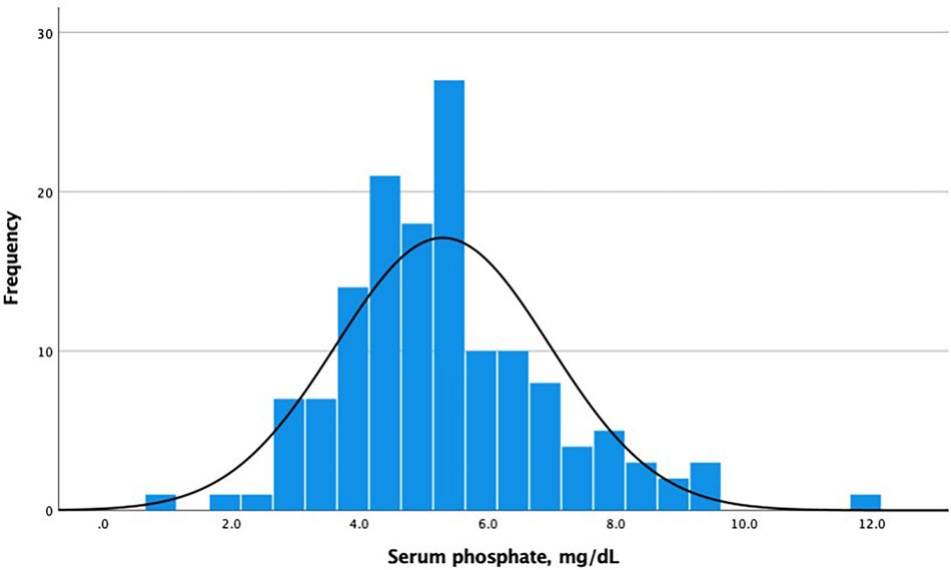
Histogram of phosphate distribution.

Although median phosphate was within the normal range (Figure 1), hyperphosphatemia was found in 51 patients (37.2%). Sex distribution, presence of diabetes, and dialysis modality were similar between patients with and without hyperphosphatemia (p > 0.05 for all comparisons). Prescription of sevelamer did not differ between patients with and without hyperphosphatemia (80.8% vs. 67.0%, p = 0.078). Phosphate levels correlated directly with serum creatinine (r = 0.471, p = 0.0001), PTH (r = 0.276, p = 0.001) and inversely with age (r = −0.250, p = 0.003).

Sevelamer hydrochloride and calcium (Ca) carbonate were prescribed as P binders to 70.8% and 10.2% of patients, respectively. Another 10.2% of patients were prescribed calcium carbonate to treat hypocalcemia following parathyroidectomy. These patients were not included in the analysis. There was no difference in P binder prescription across dialysis modalities (75.8%, 83.3%, and 75.7% of patients on conventional, short daily hemodialysis, and peritoneal dialysis, respectively, p = 0.839). The same was observed when analyzing the use of sevelamer (p = 0.818) or Ca carbonate alone (p = 0.211).

All patients on Ca carbonate were taking at least 80% of their prescribed pills. Regarding sevelamer, the medication had not been taken as prescribed by most patients (68.4%). Agreement between the prescribed dose and the dose taken was observed in 41.6% of patients, whereas 46.0% and 12.4% took a lower or higher dose than prescribed, respectively. Differences between the number of pills prescribed and taken are shown in Table 2.

**Table 2:** Agreement between number of sevelamer hydrochloride pills prescribed and in use

Pills taken per day	Number of pills prescribed per day												
	0 tablets N = 32	1 tablet N = 1	2 tablets N = 16	3 tablets N = 15	4 tablets N = 5	5 tablets N = 2	6 tablets N = 30	7 tablets N = 1	8 tablets N = 0	9 tablets N = 35	12 tablets N = 0
0 tablets	**26**	0	7	5	0	0	2	0	0	1	0
1 tablet	1	**0**	4	1	0	0	1	0	0	0	0
2 tablets	2	1	**2**	3	1	0	0	0	0	2	0
3 tablets	1	0	1	**6**	1	1	6	0	0	0	0
4 tablets	0	0	1	0	**1**	1	4	0	0	3	0
5 tablets	0	0	0	0	0	**0**	3	0	0	4	0
6 tablets	1	0	1	0	1	0	**12**	0	0	10	0
7 tablets	0	0	0	0	0	0	0	**1**	0	1	0
8 tablets	0	0	0	0	0	0	1	0	**0**	2	0
9 tablets	1	0	0	0	1	0	1	0	0	**10**	0
12 tablets	0	0	0	0	0	0	0	0	0	2	**0**

Notes – Data represent the number of patients. Bold cells identify agreement between the number of pills prescribed and taken per day.

**Figure 2:**
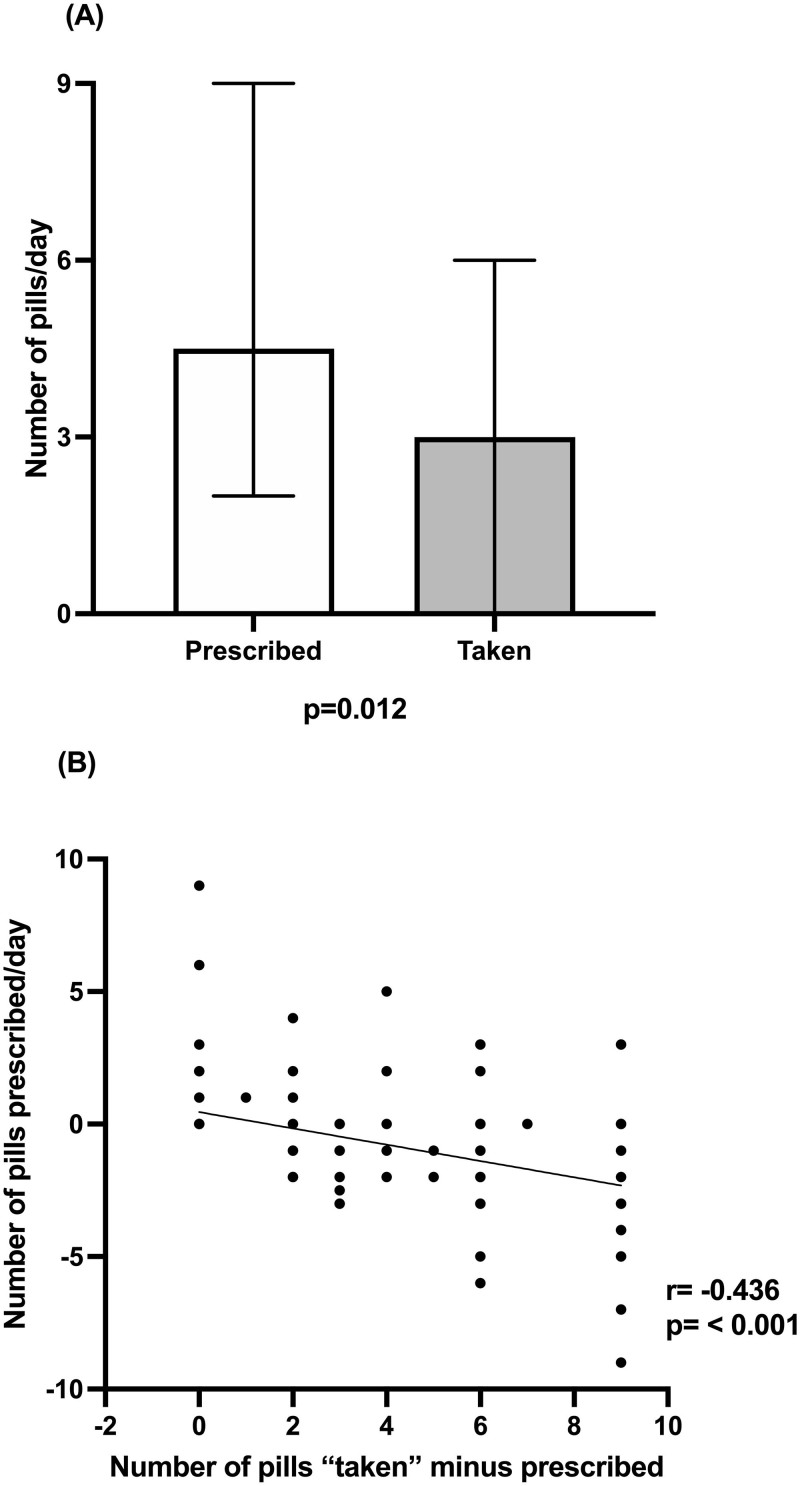
(A) Difference between the number of phosphate binder pills prescribed and taken per day. Bars represent median values and lines represent the 95% confidence interval. (B) Correlation between the number of phosphate binder pills prescribed and the difference between the number of pills taken minus prescribed.

Figure 2A shows the difference between the prescribed and “taken” dose The median prescribed dose of sevelamer per day was 4.5 (2, 9) pills and the actual dose taken was 3.0 (0, 6) pills per day. The incorrect dose varied from taking 9 pills less to even taking 9 pills more than prescribed. Figure 2B shows a correlation between the prescribed dose and the difference between the prescribed dose and the “taken” dose. P correlated with the number of pills prescribed (r = 0.368, p = 0.001) and the number of pills taken a day (r = 0.275, p = 0.001). The negative correlation showed that the higher the prescribed dose, the lower the chance of medication adherence.

Thirty-three patients were considered non-adherent to sevelamer (24.1%). These patients were mostly women, younger, with higher serum albumin and urea, and lower serum calcium (Table 1). In addition, non-adherent patients were more likely to be on conventional or short daily hemodialysis than on PD. Hyperphosphatemia was found in 35.0% of non-adherent and in 45.5% of adherent patients (p = 0.278). Logistic regression showed that female sex (HR 3.30, 95% CI: 1.39–7.84, p = 0.007) and hemodialysis (vs. PD) (HR 4.55, 95%, CI: 1.26–16.39, p = 0.021) remained independently associated with non-adherence in a model adjusted for age, calcium, and serum albumin. Similar results were obtained by modelling urea instead of albumin as the independent variable.

## Discussion

Our findings show that sevelamer was not taken correctly by most patients, and adhesion was significantly lower when a greater number of pills was prescribed. Female sex, younger age, increased levels of albumin and urea, and reduced rates of serum total calcium were associated with non-adherence. A comparison among dialysis modalities shows that patients on hemodialysis had a higher non-adherence rate.

A gold standard measurement to assess adhesion does not exist, and there is conflicting evidence regarding which measure provides the best estimate of a patient’s drug-taking behavior. According to a systematic review, most studies have used serum phosphorus levels as the method of choice to estimate adherence to P binders^11^. To estimate medication adherence, there are objective measurements such as pill count (electronic monitoring, tablet counts), subjective assessment, which is self-reported by patients or health care professionals, and pre-dialysis P levels. P levels depend on several factors such as ingestion of ultra-processed food, bone remodeling, and individual P absorption capacity, among others. In addition, those who generally do not adhere to the diet also do not adhere to the medication. The definition of non-adherence is highly variable among studies, contributing to the lack of consistency regarding phosphate binder adherence. For these reasons, the overall prevalence rate of non-adherence to medication ranges from 12.5 to 98.6%^12^.

For most patients on maintenance dialysis, current guidelines recommend that a positive calcium balance should be avoided. Calcium-based binders, when prescribed at high doses, are usually associated with hypercalcemia, adynamic bone disease, and vascular calcification. A recent meta-analysis showed a benefit of sevelamer over calcium salts in terms of mortality^13^. Concerning adherence, to our knowledge, no study has compared calcium and non-calcium-based binders. Based on these results, some researchers are currently challenging the concept that controlling serum P levels could lead to a better outcome. The ongoing studies PHOSPHATE^14^ and HILO^3^ are prospectively comparing strict control of serum P with a more liberal approach, where P binders will be prescribed only when serum P is above 7 mg/dL. Nevertheless, while the current concept and guidelines are still valid, we should look for the best strategy to achieve the recommended P target.

In agreement with our study, a systematic review published in 2008 reported a mean non-adherence rate of 51%^15^ and an association between higher prescribed pill burden and lower adherence. A derived study from DOPPS^7^ used the question “During the last month, how often did you skip taking your phosphate binders?” and compared it with the number of prescribed pills. The data show a trend towards better adherence among patients instructed to take 1-2 pills/day compared to those who received a prescription of more than 3 pills/day. Using different methods to estimate adherence, these studies and ours reached the same conclusion.

We found that non-adherent patients were most likely younger and female. In agreement with our data, young age is one of the most frequently reported factors associated with non-adherence in the literature. Female sex, on the other hand, is not often described as a risk factor for non-adherence^11^ although a previous study has demonstrated this to be similar to our data^12^. The association between high serum albumin and urea with non-adherence might reflect a nutritional status, although this is speculative.

This study had some limitations that should be considered while interpreting the results. First, since it is an observational cross-sectional study, bias by unmeasured confounders cannot be identified and causality cannot be determined. Second, we have no data on literacy and economic status associated with medication adherence. Third, adherence was assessed using a self-reported questionnaire, which may not be accurate. Fourth, we did not examine clinical outcomes. Nevertheless, this study added to the body of literature data specifically on phosphate binders. The use of a single center reduced the bias due to different phosphate treatment protocols and dietitian counselling. Finally, since the governmental health care in Brazil (Sistema Único de Saúde – SUS) provides phosphate binders to most patients on dialysis, the current study suggests that strategies to increase adherence should be implemented to achieve better phosphate control and increase adherence. Longitudinal studies are warranted to validate our results and establish an association between phosphate binder adherence and clinical outcomes.
