# Supplementary material for: Phosphate binder in dialysis: a cross-sectional study of patients' adherence and pill burden
Source: J Bras Nefrol. 2025 Jan 20;47(1):e20240075. doi: 10.1590/2175-8239-JBN-2024-0075en (PMC11748762; doi:10.1590/2175-8239-JBN-2024-0075en)
Supplement: Translation

# Translation: Quelante de fosfato na diálise: um estudo transversal sobre adesão dos pacientes e sobrecarga de comprimidos

**DOI:** 10.1590/2175-8239-JBN-2024-0075pt

**Authors:** Brunelle Bruna Scavello Coelho Ferezin, Luiza Karla Ramos Pereira de Araújo, Carolina Marquez Lima, Hugo Abensur, Benedito Jorge Pereira, Maria Aparecida Dalboni, Rosa Maria Affonso Moyses, Rosilene Motta Elias

**Affiliations:** 1 Universidade Nove de Julho, São Paulo, SP, Brasil.; 2 Universidade de São Paulo, Hospital das Clínicas, São Paulo, SP, Brasil.

**Keywords:** Phosphate Binder, P Binder, Chronic Kidney Disease, Dialysis, Hyperphosphatemia, Sevelamer, Calcium Carbonate

## Abstract

**Introdução:**

Quelantes de fosfato (P) estão entre os medicamentos mais comumente prescritos para controlar níveis de P em pacientes com DRC em diálise. Ainda há escassez de dados sobre adesão aos quelantes de P, sem comparações entre modalidades dialíticas.

**Métodos:**

Acessamos fatores associados à adesão ao quelante de P entre pacientes em diálise em um hospital universitário. A adesão foi calculada como a razão entre número de comprimidos tomados por dia, conforme relatado, e número de comprimidos prescritos. Pacientes foram considerados não aderentes se a adesão fosse ao menos 20% menor ou 30% maior do que o prescrito.

**Resultados:**

Os pacientes (N = 137) eram jovens, predominantemente mulheres, em diálise por um período mediano de 53 meses. Sevelamer e carbonato de cálcio foram prescritos como quelantes de P para 70,8% e 10,2% dos pacientes, respectivamente, sem diferença entre modalidades dialíticas (p = 0,839). P correlacionou-se com número de comprimidos prescritos (r = 0,368; p = 0,001) e número de comprimidos tomados por dia (r = 0,275; p = 0,001). Encontramos hiperfosfatemia em 52 pacientes (36,4%). A adesão ao carbonato de Ca e sevelamer foi de 100% e 68,4%, respectivamente. Pacientes não aderentes eram mulheres, mais jovens, com albumina e ureia séricas mais elevadas e menor cálcio sérico. A regressão logística mostrou que sexo feminino (HR 3,30; IC 95%: 1,39–7,84; p = 0,007) e hemodiálise *vs*. diálise peritoneal (HR 4,55; IC 95%: 1,26–16,39; p = 0,021) permaneceram independentemente associados ao comportamento de não adesão.

**Conclusão:**

O presente estudo sugere que estratégias para aumentar a adesão devem ser implementadas. A relação entre adesão ao quelante de fosfato e melhores desfechos merece investigação mais aprofundada.

## Introdução

Pacientes com doença renal crônica em diálise de manutenção (DRC5D) apresentam alto risco de hiperfosfatemia, uma complicação que requer aconselhamento dietético, diálise intensiva e o uso de quelantes de fosfato (P), os quais estão entre os medicamentos mais comumente prescritos para tais pacientes. De fato, o tratamento da hiperfosfatemia é oneroso e metade dos medicamentos prescritos por dia são, geralmente, quelantes de P^1^. As diretrizes clínicas recomendam a redução dos níveis de P para valores dentro da faixa normal e a restrição de quelantes de P à base de cálcio^2^. No entanto, estudos em andamento estão desafiando esse conceito clássico^3^.

Estudos sobre adesão medicamentosa na população em geral mostraram que 50% dos pacientes não tomam seus medicamentos conforme prescrito^4,5,6^. De acordo com o *Dialysis Outcomes and Practice Patterns Study*, somente 45% dos pacientes em diálise relataram ter tomado todos os quelantes de P prescritos no mês anterior^7^. Haynes afirmou que “o aumento da eficácia das intervenções de adesão pode ter um impacto muito maior na saúde da população do que qualquer melhoria em um tratamento médico específico”^4^. Como níveis mais elevados de P sérico têm sido associados a um maior risco cardiovascular e mortalidade^8^, a adesão ao quelante de P é uma questão importante. Esse conceito foi reforçado pela observação de uma relação inversa entre a sobrecarga de comprimidos de quelante de P e o controle do P sérico^8 9,10^.

Embora a adesão a medicamentos em geral esteja bem documentada na literatura, ainda há uma escassez de dados sobre a adesão aos quelantes de P. Além disso, não há comparação entre as modalidades de diálise sobre esse assunto. Portanto, este estudo teve como objetivo avaliar os fatores associados à adesão ao quelante de P entre pacientes em diversas modalidades de diálise (diálise peritoneal, hemodiálise convencional e hemodiálise curta diária) em um único hospital universitário no Brasil.

## Métodos

Este foi um estudo transversal que incluiu pacientes adultos (idade ≥ 18 anos) em diálise de manutenção no Hospital das Clínicas HCFMUSP, Universidade de São Paulo, Brasil. Foram convidados a participar os pacientes em hemodiálise (HD) três vezes por semana, HD curta diária ou diálise peritoneal (DP). Todos os pacientes em diálise no primeiro dia de julho de 2021 receberam o convite para participar.

Os dados clínicos, laboratoriais e demográficos foram coletados dos prontuários e incluíram idade, sexo, raça, presença de diabetes, etiologia da doença renal e modalidade de diálise. As variáveis bioquímicas de interesse foram albumina sérica (RR 3,4–4,8 g/dL), cálcio total (RR 8,4–10,2 mg/dL), fosfatase alcalina (intervalo de referência, RR 35–104 UI/L para mulheres e 40–129 UI/L para homens), fosfato (RR 2,7–4,5 mg/dL), paratormônio (PTH) (imunoensaio por quimioluminescência, RR 15–65 pg/mL) e 25(OH)-vitamina D (imunoensaio químico, RR 15–65 pg/mL). O hiperparatireoidismo foi definido como PTH > 300 pg/mL, a hipovitaminose D como níveis < 30 ng/dL e a hiperfosfatemia como níveis > 5,5 mg/dL. Foram avaliados os resultados laboratoriais mensais de rotina. Todas as análises foram realizadas pelo mesmo laboratório.

O protocolo do estudo foi aprovado pelo Comitê de Ética Local do HCFMUSP (#45163715.4.0000.0068).

### Adesão

A adesão autorrelatada foi determinada por meio da seguinte pergunta feita a cada paciente: “As pessoas não tomam seus medicamentos por diversos motivos. Quantos comprimidos você costuma tomar por dia?” Não especificamos um período de tempo para a resposta. As respostas a essa pergunta foram utilizadas para calcular a adesão autorrelatada por meio da equação: número relatado de comprimidos tomados por dia / número prescrito de comprimidos conforme a bula × 100. Se um determinado paciente informou que geralmente toma 4 comprimidos por dia para um medicamento prescrito como 2 comprimidos por dia, a adesão autorrelatada foi calculada como 200%. Os pacientes foram considerados não aderentes se a adesão autorrelatada fosse pelo menos 20% menor ou 30% maior do que a prescrita.

### Análise Estatística

Os dados contínuos são apresentados como média ± desvio padrão ou mediana (percentis 25 e 75), conforme apropriado. As variáveis categóricas são apresentadas como frequência. As comparações entre pacientes aderentes e não aderentes foram realizadas utilizando o teste t de Student ou os testes U de Mann-Whitney para variáveis com distribuição normal e não normal, respectivamente. A análise de regressão logística foi realizada para testar os fatores independentes associados à não adesão. As variáveis independentes, selecionadas a partir da análise univariada (p < 0,05), foram introduzidas uma de cada vez no modelo e incluíram idade, cálcio, modalidade de diálise (HD *vs.* PD), albumina e ureia. Como a ureia e a albumina apresentaram colinearidade, não foram incluídas no mesmo modelo. Consideramos significativos os valores de p < 0,05. Todas as análises foram realizadas utilizando o SPSS versão 26 (SPSS Inc. Chicago, IL).

## Resultados

A adesão pôde ser avaliada em 137 dos 144 pacientes triados (95,1%). Nenhum paciente se recusou a participar, 2 pacientes foram transferidos para outra unidade de diálise antes da avaliação e 5 pacientes não foram incluídos devido à falta de compreensão (2 com comprometimento mental e 3 cuja medicação era administrada por uma terceira pessoa que não pôde ser contatada durante o estudo). Conforme mostrado na Tabela 1, os pacientes eram relativamente jovens, majoritariamente mulheres caucasianas, em diálise por um período mediano de 53 meses, com baixa prevalência de diabetes. A maioria dos pacientes estava em hemodiálise de manutenção.

**Tabela 1:** Características dos pacientes

Variável	Todos N = 137	Não aderente N = 33	Aderente N = 104	P
Sexo feminino, n (%)	66 (48.2)	23 (69.7)	43 (41.3)	**0.005**
Diabetes, n (%)	22 (16.1)	17 (16.3)	5 (15.2)	0.871
Idade, anos	47 ± 17	42 ± 13	48 ± 17	**0.047**
Caucasiana	100 (73.0)	76 (73.1)	24 (72.7)	0.979
Etiologia da doença renal, n (%)	20 (14.6)	5 (15.1)	15 (14.4)	
Diabetes	17 (12.4)	3 (9.1)	14 (13.5)	
Nefrosclerose	39 (28.5)	8 (24.2)	31 (29.8)	
Glomerulonefrite	16 (11.7)	5 (15.1)	11 (10.6)	
Doença intersticial	14 (10.2)	4 (12.1)	10 (9.6)	
LES	32 (23.3)	9 (27.3)	23 (22.1)	
Outra/desconhecida				
Tempo de diálise, meses	50 (22, 92)	105 (71, 168)	50 (18, 84)	0.868
Modalidade de diálise, n (%)	89 (65.0)	27 (81.8)	62 (59.6)	**0.033**
Convencional 3 vezes/semana	12 (8.7)	3 (9.1)	9 (8.7)	
Curta diária	36 (26.3)	3 (9.1)	33 (31.7)*	
Diálise peritoneal				
Albumina sérica, mg/dL	4.0 ± 0.4	4.2 ± 0.3	4.0 ± 0.4	**0.048**
Cálcio total, mg/dL	8.9 ± 0.8	8.7 ± 0.8	8.9 ± 0.8	**0.036**
Fosfato, mg/dL	5.3 ± 1.7	5.7 ± 1.9	5.2 ± 1.6	0.103
Hiperfosfatemia, n (%)	51 (37.2)	15 (45.5)	36 (34.6)	0.278
Fosfatase alcalina UI/L	104 (76, 154)	105 (71, 168)	104 (84, 139)	0.986
Paratormônio, pg/mL	333 (193, 597)	333 (193, 585)	332 (165, 744)	0.740
Hiperparatireoidismo, n (%)	76 (55.5)	17 (51.5)	59 (56.7)	0.558
25(OH)-vitamina D, ng/dL	33.6 ± 10.5	32.9 ± 10.7	33.8 ± 10.5	0.648
Hipovitaminose, n (%)	48 (35.0)	12 (36.4)	36 (34.6)	0.849
Hemoglobina, g/dL	11.0 ± 1.7	10.9 ± 1.5	11.0 ± 1.7	0.698
Ureia, mg/dL	43 (30, 95)	47 (31, 104)	38 (28, 50)	**0.046**
Creatinina, mg/dL	9.2 ± 3.0	10.0 ± 3.2	9.0 ± 2.9	0.060

Abreviatura – LES: lúpus eritematoso sistêmico. Notas – Os dados são expressos como média ± DP ou mediana (25, 75), a menos que especificado de outra forma. *Pós-teste p < 0,05.

Embora a mediana do fosfato estivesse dentro da faixa normal (Figura 1), a hiperfosfatemia foi encontrada em 51 pacientes (37,2%). A distribuição por sexo, a presença de diabetes e a modalidade de diálise foram semelhantes entre os pacientes com e sem hiperfosfatemia (p > 0,05 para todas as comparações). A prescrição de sevelamer não diferiu entre os pacientes com e sem hiperfosfatemia (80,8% *vs.* 67,0%; p = 0,078). Os níveis de fosfato se correlacionaram diretamente com a creatinina sérica (r = 0,471; p = 0,0001), PTH (r = 0,276; p = 0,001) e inversamente com a idade (r = −0,250; p = 0,003).

**Figura 1:** Histograma da distribuição de fosfato.

O cloridrato de sevelamer e o carbonato de cálcio (Ca) foram prescritos como quelantes de P para 70,8% e 10,2% dos pacientes, respectivamente. Outros 10,2% dos pacientes receberam prescrição de carbonato de cálcio para tratar a hipocalcemia após a paratireoidectomia. Esses pacientes não foram incluídos na análise. Não houve diferença na prescrição de quelante de P entre as modalidades de diálise (75,8%, 83,3% e 75,7% dos pacientes em hemodiálise convencional, hemodiálise curta diária e diálise peritoneal, respectivamente; p = 0,839). O mesmo foi observado ao analisar o uso de sevelamer (p = 0,818) ou carbonato de cálcio isolado (p = 0,211).

Todos os pacientes em uso de carbonato de Ca estavam tomando pelo menos 80% dos comprimidos prescritos. Em relação ao sevelamer, a medicação não havia sido tomada conforme prescrito pela maioria dos pacientes (68,4%). A concordância entre a dose prescrita e a dose tomada foi observada em 41,6% dos pacientes, enquanto 46,0% e 12,4% tomaram uma dose menor ou maior do que a prescrita, respectivamente. As diferenças entre o número de comprimidos prescritos e tomados são apresentadas na Tabela 2.

**Tabela 2:** Concordância entre o número de comprimidos de cloridrato de sevelamer prescritos e em uso

Comprimi dos tomados por dia	Número de comprimidos prescritos por dia												
	0 comprimi dos N = 32	1 comprimi dos N = 1	2 comprimi dos N = 16	3 comprimi dos N = 15	4 comprimi dos N = 5	5 comprimi dos N = 2	6 comprimi dos N = 30	7 comprimi dos N = 1	8 comprimi dos N = 0	9 comprimi dos N = 35	12 comprimi dos N = 0
0 comprimi dos	**26**	0	7	5	0	0	2	0	0	1	0
1 comprimi dos	1	**0**	4	1	0	0	1	0	0	0	0
2 comprimi dos	2	1	**2**	3	1	0	0	0	0	2	0
3 comprimi dos	1	0	1	**6**	1	1	6	0	0	0	0
4 comprimi dos	0	0	1	0	**1**	1	4	0	0	3	0
5 comprimi dos	0	0	0	0	0	**0**	3	0	0	4	0
6 comprimi dos	1	0	1	0	1	0	**12**	0	0	10	0
7 comprimi dos	0	0	0	0	0	0	0	**1**	0	1	0
8 comprimi dos	0	0	0	0	0	0	1	0	**0**	2	0
9 comprimi dos	1	0	0	0	1	0	1	0	0	**10**	0
12 comprimi dos	0	0	0	0	0	0	0	0	0	2	**0**

Notas – Os dados representam o número de pacientes. As células em negrito identificam a concordância entre o número de comprimidos prescritos e os tomados por dia.

A Figura 2A mostra a diferença entre a dose prescrita e a dose “tomada” A dose mediana prescrita de sevelamer por dia foi de 4,5 (2, 9) comprimidos, enquanto a dose real tomada foi de 3,0 (0, 6) comprimidos por dia. A dose incorreta variou desde tomar 9 comprimidos a menos até tomar 9 comprimidos a mais do que o prescrito. A Figura 2B mostra uma correlação entre a dose prescrita e a diferença entre a dose prescrita e a dose “tomada”. O P se correlacionou com o número de comprimidos prescritos (r = 0,368; p = 0,001) e o número de comprimidos tomados por dia (r = 0,275; p = 0,001). A correlação negativa mostrou que quanto maior a dose prescrita, menor a chance de adesão à medicação.

**Figura 2:** (A) Diferença entre o número de comprimidos de quelante de fosfato prescritos e tomados por dia. As barras representam os valores medianos e as linhas representam o intervalo de confiança de 95%. (B) Correlação entre o número de comprimidos de quelante de fosfato prescritos e a diferença entre o número de comprimidos tomados e o prescrito.

Trinta e três pacientes foram considerados não aderentes ao sevelamer (24,1%). Esses pacientes eram predominantemente mulheres, mais jovens, com níveis séricos mais elevados de albumina e ureia e níveis séricos mais baixos de cálcio (Tabela 1). Além disso, os pacientes não aderentes apresentaram maior probabilidade de estar em hemodiálise convencional ou hemodiálise curta diária do que em DP. A hiperfosfatemia foi encontrada em 35,0% dos pacientes não aderentes e em 45,5% dos pacientes aderentes (p = 0,278). A regressão logística revelou que o sexo feminino (HR 3,30; IC 95%: 1,39–7,84; p = 0,007) e a hemodiálise (*vs.* DP) (HR 4,55; IC 95%: 1,26–16,39; p = 0,021) permaneceram independentemente associados à não adesão em um modelo ajustado para idade, cálcio e albumina sérica. Resultados semelhantes foram obtidos ao modelar a ureia em vez da albumina como a variável independente.

## Discussão

Nossos achados mostram que o sevelamer não foi tomado corretamente pela maioria dos pacientes e a adesão foi significativamente menor quando um número maior de comprimidos foi prescrito. Sexo feminino, idade mais jovem, níveis aumentados de albumina e ureia e taxas reduzidas de cálcio total sérico foram associados à não adesão. Uma comparação entre as modalidades de diálise mostra que os pacientes em hemodiálise apresentaram uma taxa de não adesão mais elevada.

Não existe uma medida padrão-ouro para avaliar a adesão, e há evidências conflitantes sobre qual medida fornece a melhor estimativa do comportamento de consumo de medicamentos de um paciente. De acordo com uma revisão sistemática, a maioria dos estudos utilizou os níveis séricos de fósforo como o método de escolha para estimar a adesão aos quelantes de P^11^. Para estimar a adesão à medicação, existem medidas objetivas, como a contagem de comprimidos (monitoramento eletrônico, contagens de comprimidos), avaliação subjetiva, que é autorrelatada por pacientes ou profissionais de saúde, e níveis de P pré-diálise. Os níveis de P dependem de diversos fatores, como a ingestão de alimentos ultraprocessados, a remodelação óssea e a capacidade individual de absorção de P, entre outros. Além disso, aqueles que geralmente não aderem à dieta também não aderem à medicação. A definição de não adesão é altamente variável entre os estudos, o que contribui para a falta de consistência quanto à adesão ao quelante de fosfato. Por esses motivos, a taxa de prevalência geral de não adesão à medicação varia de 12,5 a 98,6%^12^.

Para a maioria dos pacientes em diálise de manutenção, as diretrizes atuais recomendam que um balanço positivo de cálcio seja evitado. Os quelantes à base de cálcio, quando prescritos em doses elevadas, geralmente estão associados a hipercalcemia, doença óssea adinâmica e calcificação vascular. Uma meta-análise recente demonstrou um benefício do sevelamer em relação aos sais de cálcio em termos de mortalidade^13^. No que diz respeito à adesão, até onde sabemos, nenhum estudo comparou quelantes à base de cálcio com aqueles que não são à base de cálcio. A partir desses resultados, alguns pesquisadores estão atualmente questionando o conceito de que o controle dos níveis séricos de P pode levar a um melhor desfecho. Os estudos em andamento PHOSPHATE^14^ e HILO^3^ estão comparando prospectivamente o controle rigoroso dos níveis séricos de P com uma abordagem mais liberal, em que os quelantes de P serão prescritos somente quando o P sérico estiver acima de 7 mg/dL. No entanto, embora o conceito e as diretrizes atuais ainda sejam válidos, devemos buscar a melhor estratégia para alcançar a meta de P recomendada.

Em concordância com nosso estudo, uma revisão sistemática publicada em 2008 relatou uma taxa média de não adesão de 51%^15^ e uma associação entre maior carga de comprimidos prescrita e menor adesão. Um estudo derivado do DOPPS^7^ usou a pergunta “Durante o último mês, com que frequência você deixou de tomar seus quelantes de fosfato?” e a comparou com o número de comprimidos prescritos. Os dados mostram uma tendência de melhor adesão entre os pacientes instruídos a tomar de 1 a 2 comprimidos/dia em comparação com aqueles que receberam uma prescrição de mais de 3 comprimidos/dia. Utilizando métodos diferentes para estimar a adesão, esses estudos e o nosso chegaram à mesma conclusão.

Constatamos que os pacientes não aderentes eram, provavelmente, mais jovens e do sexo feminino. Em conformidade com nossos dados, a idade jovem é um dos fatores mais frequentemente relatados na literatura como associado à não adesão. Por outro lado, o sexo feminino não é frequentemente descrito como um fator de risco para a não adesão^11^ embora um estudo anterior tenha demonstrado semelhança com nossos dados^12^. A associação entre níveis séricos elevados de albumina e ureia com a não adesão pode refletir um estado nutricional, embora isso seja especulativo.

Este estudo apresentou algumas limitações que devem ser consideradas ao interpretar os resultados. Em primeiro lugar, por se tratar de um estudo observacional transversal, não foi possível identificar vieses por fatores de confusão não mensurados e a causalidade não pode ser determinada. Segundo, não dispomos de dados sobre alfabetização e status econômico associados à adesão medicamentosa. Em terceiro lugar, a adesão foi avaliada por meio de um questionário de autorrelato, que pode não ser preciso. Quarto, não examinamos desfechos clínicos. No entanto, este estudo agregou ao conjunto de dados da literatura especificamente sobre quelantes de fosfato. O uso de um único centro reduziu o viés devido a diferentes protocolos de tratamento com fosfato e aconselhamento nutricional. Por fim, uma vez que o sistema de saúde governamental no Brasil (Sistema Único de Saúde - SUS) fornece quelantes de fosfato para a maioria dos pacientes em diálise, o presente estudo sugere que estratégias para aumentar a adesão devem ser implementadas para se obter um melhor controle de fosfato e maior adesão. São necessários estudos longitudinais para validar nossos resultados e estabelecer uma associação entre a adesão ao quelante de fosfato e os desfechos clínicos.
